# Monosodium Glutamate Dietary Consumption Decreases Pancreatic β-Cell Mass in Adult Wistar Rats

**DOI:** 10.1371/journal.pone.0131595

**Published:** 2015-06-29

**Authors:** Piyanard Boonnate, Sakda Waraasawapati, Wiphawi Hipkaeo, Supattra Pethlert, Amod Sharma, Carlo Selmi, Vitoon Prasongwattana, Ubon Cha’on

**Affiliations:** 1 Department of Biochemistry, Faculty of Medicine, Khon Kaen University, Khon Kaen, Thailand; 2 Department of Pathology, Faculty of Medicine, Khon Kaen University, Khon Kaen, Thailand; 3 Department of Anatomy, Faculty of Medicine, Khon Kaen University, Khon Kaen, Thailand; 4 Rheumatology and Clinical Immunology, Humanitas Clinical and Research Center, Milan, Italy; 5 Rheumatology, Allergy, and Clinical Immunology, University of California Davis, Davis, California, United States of America; Consiglio Nazionale delle Ricerche, ITALY

## Abstract

**Background:**

The amount of dietary monosodium glutamate (MSG) is increasing worldwide, in parallel with the epidemics of metabolic syndrome. Parenteral administration of MSG to rodents induces obesity, hyperglycemia, hyperlipidemia, insulin resistance, and type 2 diabetes. However, the impact of dietary MSG is still being debated. We investigated the morphological and functional effects of prolonged MSG consumption on rat glucose metabolism and on pancreatic islet histology.

**Methods:**

Eighty adult male Wistar rats were randomly subdivided into 4 groups, and test rats in each group were supplemented with MSG for a different duration (1, 3, 6, or 9 months, n=20 for each group). All rats were fed *ad libitum* with a standard rat chow and water. Ten test rats in each group were provided MSG 2 mg/g body weight/day in drinking water and the 10 remaining rats in each group served as non-MSG treated controls. Oral glucose tolerance tests (OGTT) were performed and serum insulin measured at 9 months. Animals were sacrificed at 1, 3, 6, or 9 months to examine the histopathology of pancreatic islets.

**Results:**

MSG-treated rats had significantly lower pancreatic β-cell mass at 1, 6 and 9 months of study. Islet hemorrhages increased with age in all groups and fibrosis was significantly more frequent in MSG-treated rats at 1 and 3 months. Serum insulin levels and glucose tolerance in MSG-treated and untreated rats were similar at all time points we investigated.

**Conclusion:**

Daily MSG dietary consumption was associated with reduced pancreatic β-cell mass and enhanced hemorrhages and fibrosis, but did not affect glucose homeostasis. We speculate that high dietary MSG intake may exert a negative effect on the pancreas and such effect might become functionally significant in the presence or susceptibility to diabetes or NaCl; future experiments will take these crucial cofactors into account.

## Introduction

Monosodium glutamate (MSG), a sodium salt of glutamic acid, is used as a flavor enhancer in food industry [1]. While the Food and Drug Administration (FDA) stated that MSG is safe as a flavor enhancer, its safety as a food additive remains debated. Epidemiological studies from our group and others reported the association of dietary MSG consumption with metabolic disorders such as obesity or above average weight [2, 3], arterial hypertension [4] and metabolic syndrome [5], while others reported the lack of such associations [6, 7]. Nonetheless, consistent metabolic effects of MSG have been demonstrated in animal studies. First, subcutaneous (SC) MSG injections (2mg/g body weight) given to newborn mice result in central obesity and moderate to severe microvesicular fatty changes throughout the liver parenchyma at 6 months [8]. Second, the same dose causes the elevation of fasting blood glucose levels and ultimately type 2 diabetes [9]. Third, higher doses of parenteral MSG (4 mg/g body weight) in mice cause insulin resistance as illustrated by the significant increase in plasma glucose following the oral glucose tolerance test (OGTT) and severe visceral fat accumulation [10]. Pathology in these models showed pancreatic islets hypertrophy [9], hyperplasia [11]and decreased α-, and somatostatin cells [12].

Despite these lines of evidence, the effects of dietary consumption of MSG are less clear, particularly the effect on pancreas histology in which numerous factors have been widely investigated and should be taken into account [13, 14]. We therefore investigated the effects of oral MSG supplementation on the rat pancreatic islets. We demonstrated significant changes in the pancreas as early as after one month of MSG supplementation. These changes increased with longer MSG supplementation, albeit without observable functional consequences on glucose metabolism.

## Materials and Methods

### Animals

Eighty male Wistar rats (weight 150–200g) were obtained at 5 weeks of age from the National Laboratory Animal Center, Salaya, Mahidol University, Thailand. Rats were housed in light and temperature controlled environment at the Northeast Laboratory Animal Center (NELAC) for 1 week before starting the experiment.

All procedures were performed in accordance with the guidelines of the Northeast Laboratory Animal Center (NELAC), Khon Kaen University, Thailand, and were approved (AEKKU 24/2554) by the Animal Ethics Committee of Khon Kaen University, Thailand.

### Experimental design

Rats were maintained under controlled laboratory conditions at the temperature of 25±3°C with 60±15% humidity and 12 h dark/light cycle. All rats were fed *ad libitum* with a standard rat chow pellet (Perfect Companion Group, Bangkok, Thailand) and provided drinking water purified by reverse osmosis (RO), either with or without MSG.

Eighty rats were randomly arrayed into four groups to be observed for1, 3, 6, or 9 months, with 20 rats in each group. Each group included control (n = 10) and MSG-treated (n = 10) rats. MSG-treated rats were supplemented with a commercially available 99%-pure food-grade package of MSG added to daily drinking water at the final daily dose of 2 mg/g body weight. Food intake and body weight were recorded every one and two weeks, respectively, and rats from different groups were sacrificed at 1, 3, 6, or 9 months following a 12-hour fasting by intraperitoneal Nembutal injection. Blood and pancreatic tissue were collected for functional and morphological study.

### Histology and immunohistochemistry

The pancreas tail was cut and fixed with a 10% neutral buffered formalin solution. Routinely processed paraffin-embedded tissue blocks were sliced at 4 μm thickness and sections were stained with Haematoxylin&Eosin (H&E) and observed under a light microscope (Primo Star, Zeiss). Prussian blue and Masson’s trichrome staining were used to determine hemosiderin deposition and fibrosis of the islets, respectively.

Immunohistochemistry was used to identify islets β-cells and 4-hydroxynonenal (4-HNE), respectively, using immunoperoxidase staining. Tissue sections were deparaffinized, rehydrated, and endogenous peroxidase and non-specific binding were blocked by 0.3% H_2_O_2_ in methanol and 3% bovine serum albumin (BSA), respectively. Sections were then incubated overnight at room temperature with a mouse monoclonal anti-insulin (dilution 1:4000, Sigma-Aldrich, USA) and a rabbit polyclonal anti-4-HNE (dilution 1:2000, Abcam, USA). The sections were washed with phosphate-buffered saline (PBS, pH 7.4), and incubated 1 h at room temperature with a goat anti-mouse IgG peroxidase antibody (dilution 1:250, Sigma-Aldrich, USA)or anti-rabbit envision^+^ HRP (horse reddish peroxidase, Sigma-Aldrich, USA), washed with phosphate-buffered saline (PBS, pH 7.4) and then stained with 3,3’-diaminobenzidine tetrahydrochloride (DAB) (Sigma-Aldrich, USA) plus a substrate buffer solution and hydrogen peroxide.

Numbers of islets, islets sizes (μm^2^) and total pancreas areas (μm^2^) in each section were measured by Aperio Image Scope software (version 12.0.1.5027). Islets and pancreatic tissue boundaries were marked manually and all islets composed of ≥ 4 cells were marked. Sizes of islets in the control groups were pooled and 5th, 10th, 25th, 50th, 75th, 90th and 95th percentiles were calculated. The density of pancreatic islets (the number of islets per unit area mm^2^) was calculated by the total number of islets divided by total area of pancreas in each slide, according to the following formula.

$$\text{Islets density = }\frac{\text{Total number of islets}}{{\text{Total area of pancreas (mm}}^{\text{2}}\text{)}}$$

The β-cells and 4-HNE of selected islets were analyzed by Aperio Image Scope software using Positive Pixel Count Algorithm (Version 9.1). The positive immunoreactivity, which produced brown color by DAB, was digitally expressed as yellow, orange and reddish-brown color pixels in the software correlated to weak, moderate, and strongly positive, respectively, and the negative immunoreactivity was expressed as blue color pixels. The percentage of β-cells and 4-HNE were calculated as the equation below.

$$\beta \text{ - cell mass (\%) = }\frac{\text{Positive pixel count}}{\text{Total pixel count}}\times 100$$

$$\text{4 - HNE (\% positivity) = }\frac{\text{Positive pixel count}}{\text{Total pixel count}}\times 100$$

Hemorrhage and fibrosis in pancreatic islets were observed in H&E slides then confirmed with special staining. Erythrocytes leaked from capillaries, brown pigments deposition in/around the islets and presence of hemosiderin were scored from mild to severe levels. The brown pigments were confirmed by the positive staining with Prussian blue. Fibrosis was confirmed and scored by Masson’s trichrome staining. The severity of the lesions was scored for each islet. The incidence of hemorrhage/hemosiderin deposition and fibrosis in islets were calculated in each slide (20–200 islets/slide). The percentage of rats with an islet lesion was calculated by the number of rats with an islet lesion divided by the total number of rats in each group.

To evaluate the severity of the lesions of pancreatic islets, hemorrhage/hemosiderin staining and fibrosis were scored using the modified methods of Imaoka *et al*[15]. The scoring were then categorized into positive and negative categories (score 0 = negative, score 1–3 = positive). Percent incidence of hemorrhage, hemosiderin and fibrosis was calculated as the equation below.

$$\text{\% incidence of lesions = }\frac{\text{Number of islets with lesions}}{\text{Total number of islets}}\times 100$$

### Insulin levels and oral glucose tolerance test (OGTT)

Insulin levels were determined at 1, 3, 6, and 9 months prior to sacrifice using radio immunoassay (RIA) kit following the manufacturer's protocol (Millipore, USA). OGTT were performed at 9 months. After12-h overnight fasting, a glucose solution (4 g/kg body weight) was fed to the rats. Blood samples were collected from the tail vein and glucose levels were measured at 0, 30 and 120 minutes after administration of glucose.

### Statistical analyses

For statistical analyses, IBM SPSS statistics software ver.19.0.2 for Windows (KKU network license) was used. All variables are presented as mean ± SD except for insulin levels (mean ± SEM). Data between control and MSG-treated groups were compared by Student’s *t*-test. *P* values ≤0.05 were considered statistically significant.

## Results

### Body weight, food and water intake

**Fig 1:**
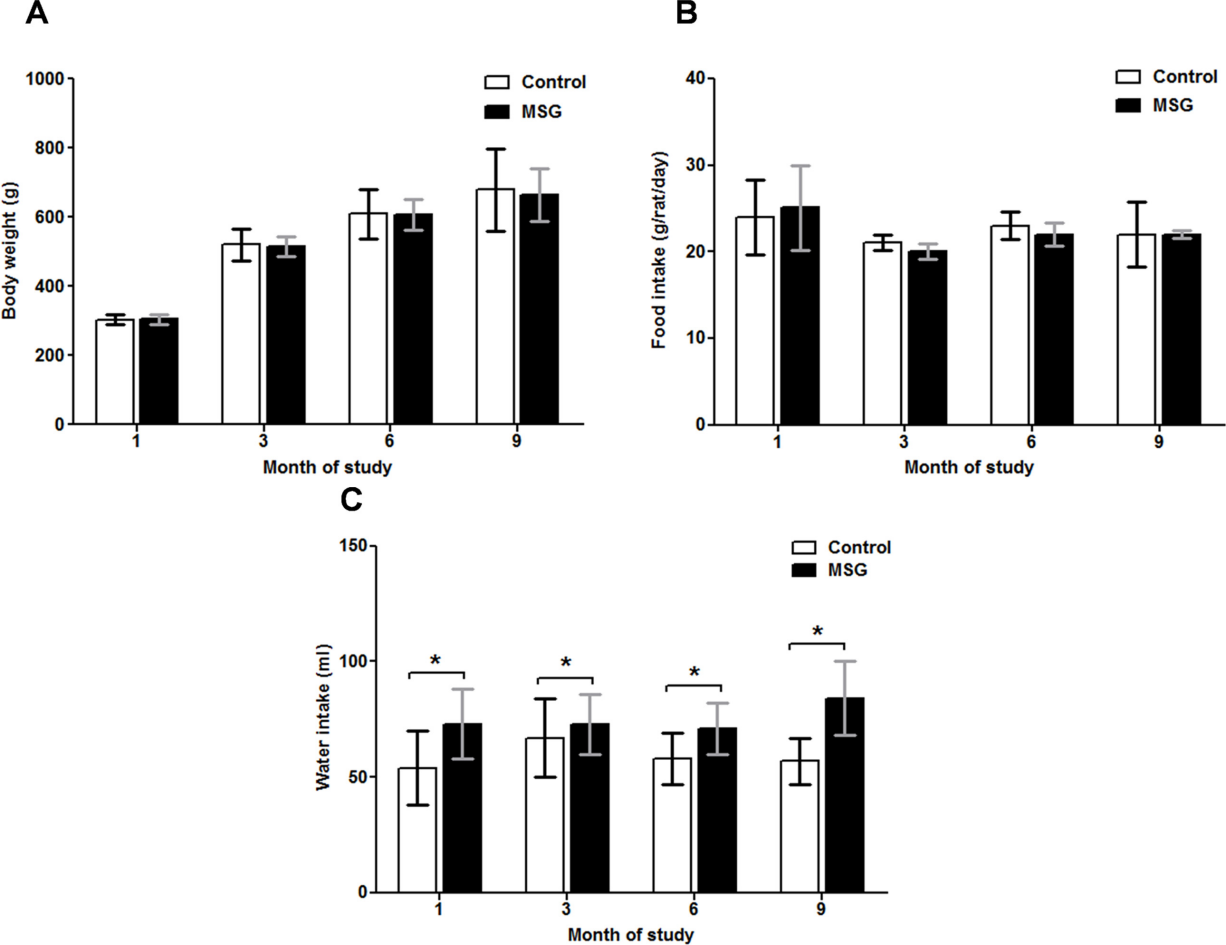
Body weight (panel A), food (panel B) and water (panel C) intake in the control and MSG-treated groups at 1, 3, 6, and 9 months of study. * *P*<0.05.

Body weight and food consumption were similar in MSG-treated and untreated control rats at 1, 3, 6 and 9 months (Fig 1A and 1B), while water intake was significantly higher in the MSG-treated groups compared to controls throughout the study period(*P* < 0.05) (Fig 1C).

### Pancreas histopathology

**Fig 2:**
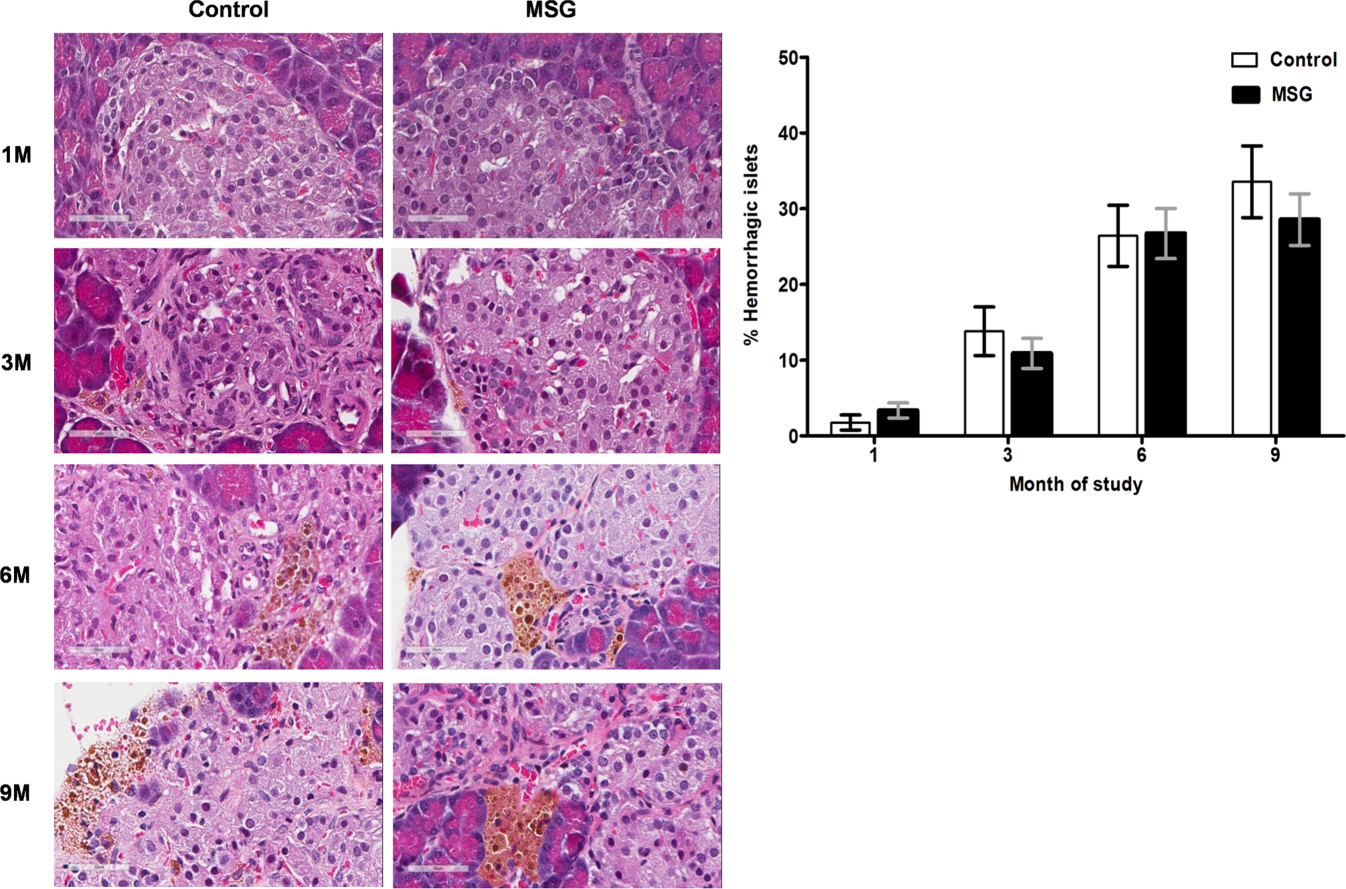
Representative histology (left) and prevalence (right) of islets hemorrhage/hemosiderin deposits in the control and MSG-treated group at 1, 3, 6, and 9 months (x400).

**Fig 3:**
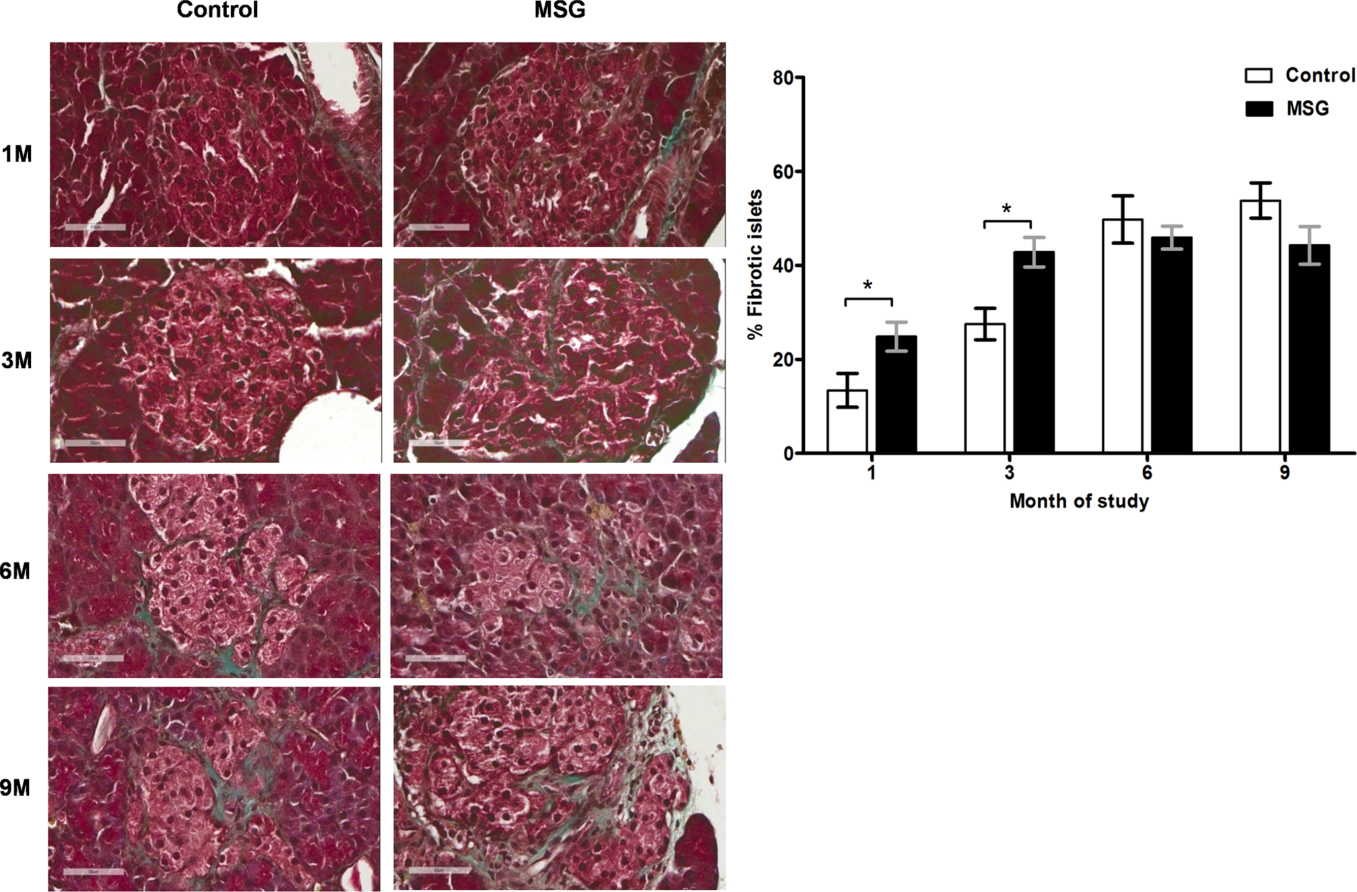
Representative histology (left) and prevalence (right) of islet fibrosis in control and MSG-treated group at 1, 3, 6, and 9 months (Masson’s trichrome staining, x400).

**Fig 4:**
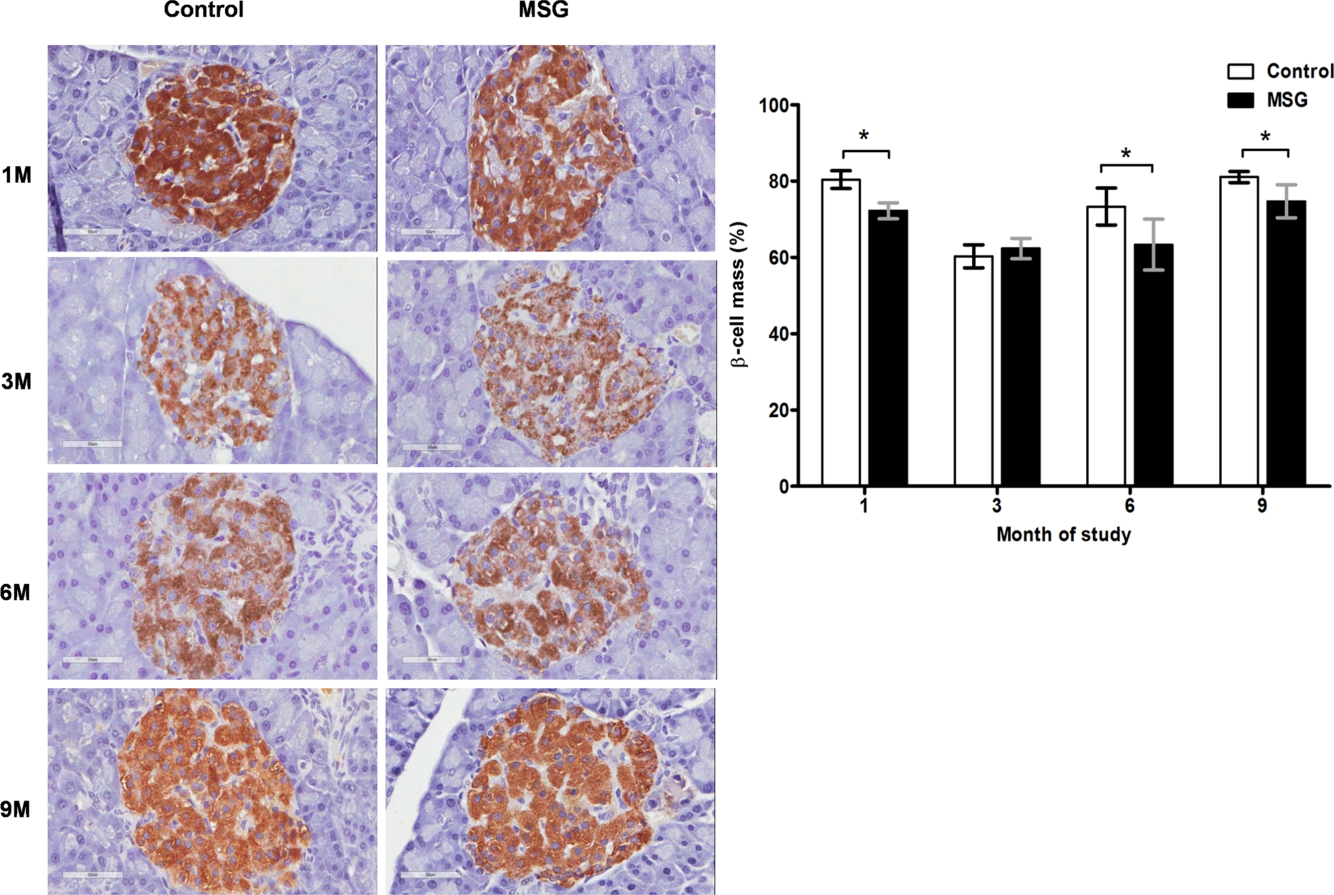
Representative immunohistochemistry (left) and prevalence (right) of insulin staining in control versus MSG-treated groups at 1, 3, 6, and 9 months (x400). * *P*<0.05.

**Fig 5:**
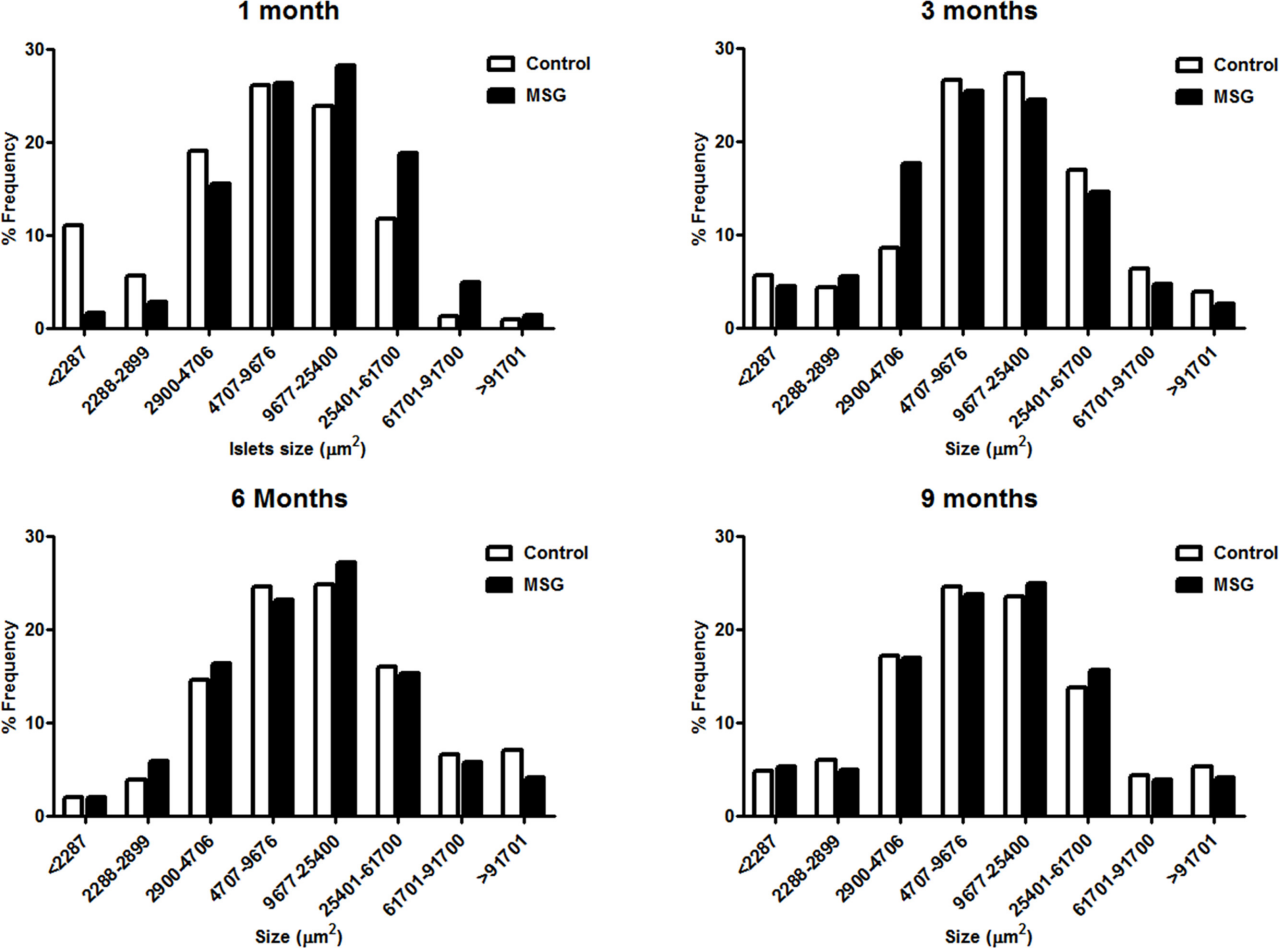
Islet size (um^2^) distribution in control and MSG-treated groups at 1, 3, 6, and 9 months.

**Fig 6:**
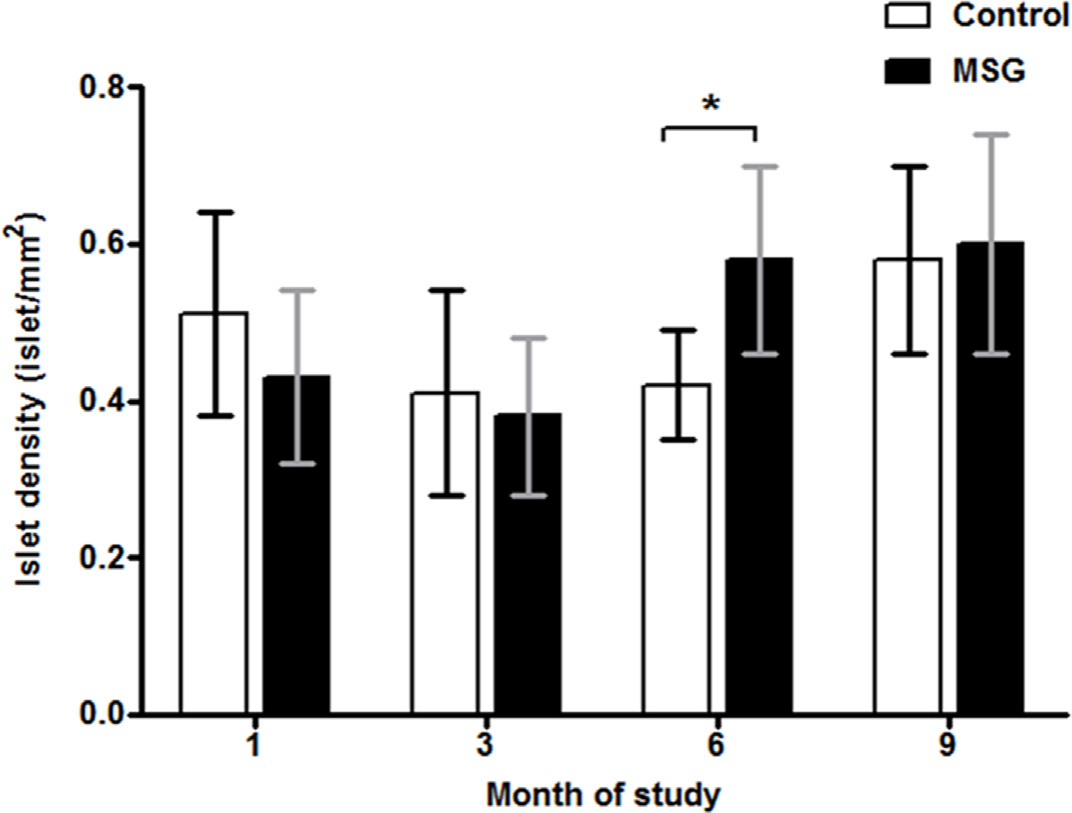
Islet density in control and MSG-treated groups at 1, 3, 6, and 9 months.

**Fig 7:**
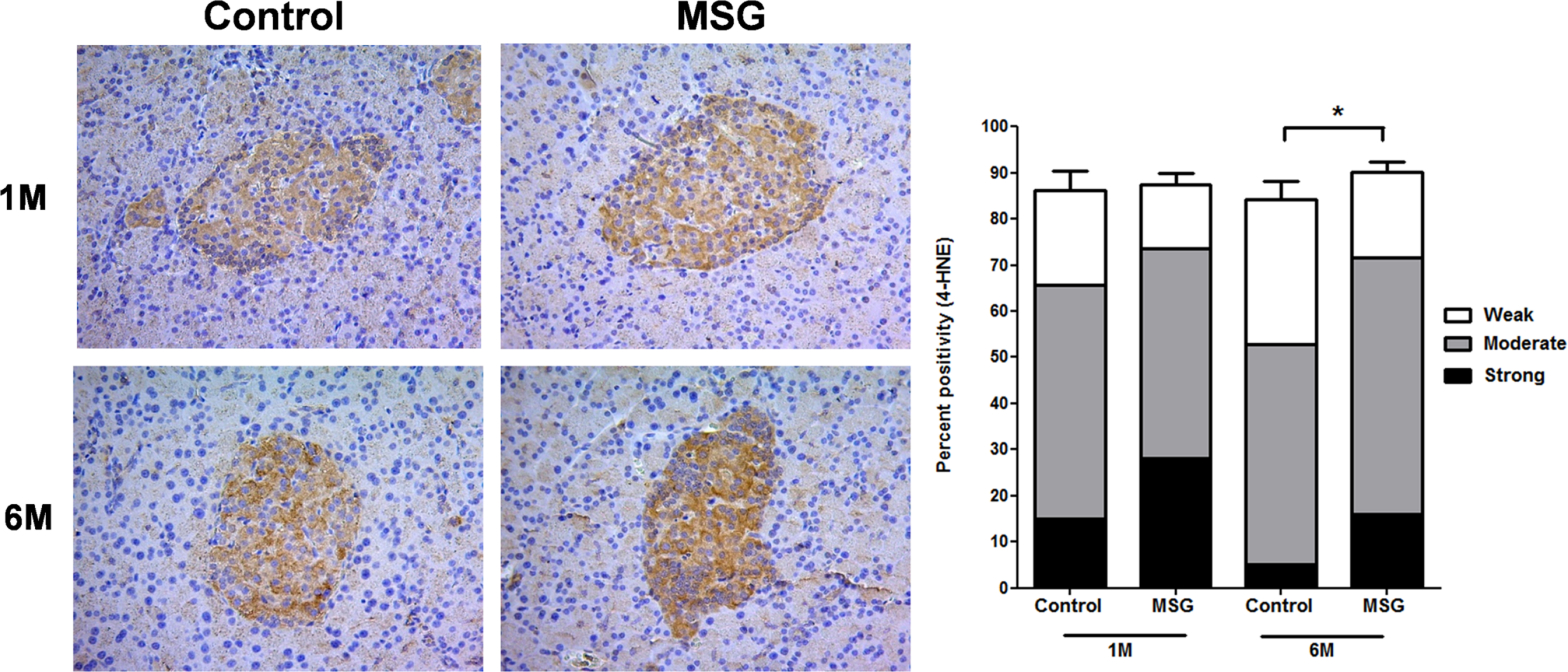
Representative immunohistochemistry (left) and prevalence (right) of 4-HNE staining which was divided into 3 categories (weak, moderate, and strong) based on its intensity in control versus MSG-treated groups at 1 and 6 months (x200). * *P* = 0.001.

Hemorrhages in the pancreatic islets were observed in both control and MSG-supplemented groups in 4/10 and 7/10 rats at 1 month, 8/10 and 9/10at 3 months, 10/10 in both groups at 6 and 9 months, respectively (Fig 2). However, differences were not significant between the groups throughout the study period. Pancreatic islet fibrosis also increased with age in both groups but was more prominent in the MSG-treated group at 1 and 3 months of the study(*P*< 0.0001) (Fig 3). The β-cell areas in the pancreatic islets were significantly lower in the MSG-treated group compared with controls at 1, 6 and 9 months (*P*< 0.05) (Fig 4). Pancreatic islet size distribution in the MSG-treated group was significantly higher than that of the control groups at 1 month (Fig 5) but was similar in both groups throughout the remaining period. The islet density of MSG-treated group was significantly higher compared to controls at 6 months (0.58 ± 0.12 vs. 0.42 ± 0.07; *P* <0.05), but such difference was not seen in any other time points (Fig 6). The intensity of immune-staining of 4-HNE, an oxidative stress marker, in the pancreatic islets of the MSG-treated group was slightly (but not significantly) higher at 1 month and was significantly higher at 6 months compared to the control group (*P* = 0.001) (Fig 7).

### Pancreatic functional tests

**Fig 8:**
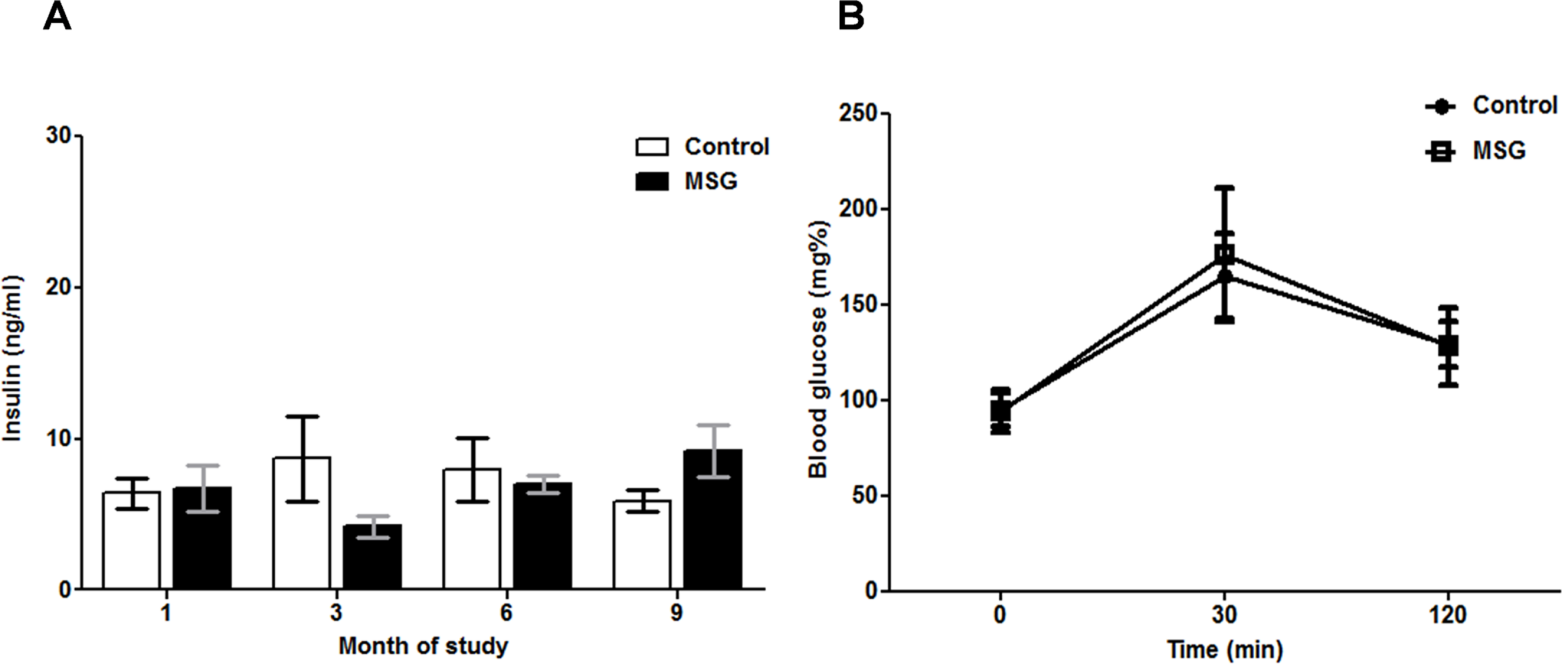
Pancreatic function in control and MSG-treated groups as measured by (A) insulin levels at 1, 3, 6, and 9 months (mean ± SEM) and (B) oral glucose tolerance test (OGTT) at 9 months(mean ± SD).

There was no significant difference in serum insulin levels between control and MSG-treated groups at anytime point examined (Fig 8A). Similarly, OGTT performed at 9 months of study showed no difference in glucose levels between control and MSG-treated groups (Fig 8B).

## Discussion

Whether MSG intake is a causative factor in epidemics of metabolic syndrome remains largely debated, particularly since there are epidemiological and experimental data both for and against this view. Indeed, metabolic syndrome involves insulin resistance, and pancreatic changes should be expected. Animal studies have demonstrated that parenteral MSG causes various changes in pancreatic islets such as hypertrophy [9], hyperplasia [11], decrease in acinar cells, α-cells and somatostatin cells [12] and increase in fibrosis [16]. However, these conditions do not represent the scenario of oral supplementation and for this reason we herein investigated the effects of oral MSG on the histomorphological and functional alterations of the rat pancreatic islets.

We report for the first time that daily consumption of dietary MSG decreases the pancreatic β-cell mass in adult rats, similar to what has been observed following MSG injection in newborn mice [12, 16]. However, the mechanism of the two might be different. The mechanism of MSG-induced β-cell loss in this study remains unclear, but the increased oxidative stress by MSG in β-cells is one possibility [17],which was supported by the increase of 4-HNE levels in the pancreatic islets in the MSG-treated group in this study. Alloxan [18], streptozotocin (STZ)[18], and ethanol [19]may induce β-cells loss through different mechanisms. First, in the presence of intracellular thiols, especially glutathione, alloxan generates reactive oxygen species (ROS) in a cyclic redox reaction with its reduction product, dialuric acid. Autoxidation of dialuric acid generates ROS, which is in turn responsible for the β-cells necrosis [18]. Second, STZ is separated into its glucose and methylnitroso-urea moietyinside β-cells and, due to its alkylating properties, the latter modifies biological macromolecules, fragments DNA and destroys the β-cells, causing a state of insulin-dependent diabetes [18]. Finally, chronic ethanol consumption decreases the expression and inactivation of glucokinase, a key glycolytic enzyme,by tyrosine nitration, leading to pancreatic β-cell apoptosis [20].

We also observed islet hypertrophy in the MSG group at 1 month, similar to what reported in mice receiving MSG injections at birth [9, 10]. Pancreatic islet hypertrophy is generally observed in obesity and is secondary to high fat diets [21], chronic glucose infusion [22], and partial pancreatectomy [23] followed by β-cell regeneration [24]. We speculate that similar mechanisms may cause islet hypertrophy after oral MSG treatment. In agreement with this observation we spot a higher density of pancreatic islets in MSG-treated rats compared to control at 6 months and expect that such density may increase the β-cell mass during regeneration. This has been reported in mice following islet [25] and β-cell [26] injury and may represent a reactive phenomenon in our experimental setting.

Daily consumption of MSG had minimal effects on islet hemorrhage, which in our study increased with age regardless of MSG. This result is consistent with previous studies that showed spontaneous hemorrhage in older males [15]. Nonetheless, the frequency of hemorrhage was higher with MSG consumption at 1 and 3 months and later reached a plateau, eventually affecting 100% of animals. It is possible that the islet hemorrhage and hemosiderin deposition may be responsible for fibrosis that we and other groups have observed. In a previous study, male Wistar rats fed with Busulfan, an alkylating anticancer drug, developed islet hemorrhages and islet fibrosis [27]. Fibrosis is a physiological healing process that occurs after tissue damage/injury [28, 29]. In our study, fibrotic lesions were significantly more frequent in the MSG-treated rats at 1 and 3 months compared to that in the controls, resembling what was reported in rats receiving parenteral MSG at birth [16]. Similar to the hemorrhagic pattern, fibrotic islets increased with age and aging overcome the MSG effect at later time points.

Based on the observed pathology of the pancreas, we were surprised to observe no changes of insulin levels or glucose tolerance during MSG supplementation. We assume that the functional impairment may require a second hit or a separate factor for susceptibility. Our assumption based on the previous studies was that MSG was converted into 2 major products, glucose and lactate, within two hours after ingestion [30]. Therefore, MSG consumption during meals might increase postpandrial glucose levels and then stimulate insulin release. Indeed, MSG has been shown to have an effect on insulin release. In human studies, serum insulin levels tended to be higher in MSG-treated participants compared to controls and the level of serum insulin correlated well with that of glutamate [31]. This was supported by a molecular study that showed that glutamate transporters are expressed in pancreatic β-cells and play a role in the regulation of insulin secretion [32]. Moreover, glutamate itself induces pancreatic β-cell damage [17]. A high level of extracellular glutamate impaired the uptake of cysteine, a precursor of glutathione synthesis, by inhibiting Glu-Cys antiporter in pancreatic β-cells, leading to oxidative stress and cell death. We also observed the β-cell loss in this study. It is possible that the dose or length of time used in this study is insufficient to induce this effect, or that the observed effects may become significant only in the presence of a genetic susceptibility or a pre-existing chronic condition. The use of dietary induced obese rats or genetically obese and diabetic Zucker rats will be useful to further evaluate the effect of MSG on glucose homeostasis, pancreatic function and histology [33]. Similarly, we are convinced that a deeper study of the functional features of islet β-cells [34, 35]induced by MSG would be a mechanistic development of our observation but this possibility was not included in the prior aims and scopes of this study.

Of note, the dose given to rats is 5–6 times higher than the current average estimate of MSG consumption in Asia (4 g/day)[36] and 2–3 times higher than what is considered as safe (150 mg/kg, 10 g/day for a 70 kg man)[37]. However, the dose given to rats in this study is closer to the daily amount recorded in a subgroup of subjects in our previous study in the Thai population (9–14 g/day)[5]

The higher intake of water observed in the MSG-treated rats is probably not related to the function of pancreatic tissue, but rather to renal tissue. MSG-treated group excreted a greater volume of urine and sodium per day compared to controls [38]. The higher water intake is likely a response by the rats to balance the higher volume loss via urine excretion. It should be noted that in the rodent model, the effect of MSG on obesity and/or metabolic syndrome during adulthood is different from that in the perinatal period of development, a time when the blood-brain barrier is immature and most vulnerable to toxicity in rodents [39, 40]. At high concentration, MSG is neurotoxic and destroys cells in the arcuate hypothalamic nuclei, which is a large hypothalamic area responsible for controlling body weight and energy balance [39]. In contrast, the effects induced by MSG-consumption at adulthood are likely to occur by a different mechanism.

Finally, we are well aware that numerous additional factors may play a pivotal role in the development or protection of the phenotype observed. First, we recognize that the presence of NaCl should be addressed in the future by dedicated experiments with equimolar NaCl solutions in animal models [13, 41]. Second, the individual susceptibility to diabetes is necessarily a key factor and the use of strains prone to metabolic disturbances such as genetically determined [42]or diet induced [33]obese rat diabetes will prove a much needed proof of this hypothesis, despite the variable characteristics of these models [43]. Third, additional factors such as protein intake or drug treatments should also be accounted for in future experiments [14, 44]. Nonetheless, the experimental design to discriminate the effect of all these specific factors must be prospective and could not be completed in a retrospective fashion.

## Conclusion

We demonstrated here that daily MSG consumption increases pancreatic β-cell loss, but this does not affect baseline insulin levels or glucose tolerance in normal adult rats. While these results support the argument that MSG is likely to be safe for use in the healthy population, these results also suggest that significant adverse effects may be seen in subjects with genetic susceptibility to diabetes or with a preexisting chronic pancreatitis. We also submit that additional factors not accounted in our experimental design include the individual genetic susceptibility and other nutritional factors which will be the objective of future studies, along with functional studies of islet cells exposed to MSG.
